# The safety and efficacy of live attenuated influenza vaccine in young children with asthma or prior wheezing

**DOI:** 10.1007/s10096-012-1595-9

**Published:** 2012-03-14

**Authors:** C. S. Ambrose, F. Dubovsky, T. Yi, R. B. Belshe, S. Ashkenazi

**Affiliations:** 1 Medical and Scientific Affairs, MedImmune, LLC, One MedImmune Way, Gaithersburg, MD 20878 USA; 2 St. Louis University School of Medicine, 1100 S. Grand Blvd., DRC-8th Floor, St. Louis, MO 63104 USA; 3 Schneider Children’s Medical Center, 14 Kaplan St., Petach Tikva, 49202 Israel; 4 Sackler Faculty of Medicine, Tel Aviv University, Ramat Aviv, 69978 Israel

## Abstract

In the European Union and Canada, an Ann Arbor strain live attenuated influenza vaccine (LAIV) is approved for use in children aged 2–17 years, including those with mild to moderate asthma or prior wheezing. The safety and efficacy of LAIV versus trivalent inactivated influenza vaccine (TIV) in children with asthma aged 6–17 years have been demonstrated. However, few data are available for children younger than 6 years of age with asthma or prior wheezing. Safety and efficacy data were collected for children aged 2–5 years with asthma or prior wheezing from two randomized, multinational trials of LAIV and TIV (*N* = 1,940). Wheezing, lower respiratory illness, and hospitalization were not significantly increased among children receiving LAIV compared with TIV. Increased upper respiratory symptoms and irritability were observed among LAIV recipients (*p* < 0.05). Relative efficacies were consistent with the results observed in the overall study populations, which demonstrated fewer cases of culture-confirmed influenza illness in LAIV compared with TIV recipients. Study results support the safety and efficacy of LAIV among children aged 2–17 years with mild to moderate asthma or a history of wheezing. Data regarding LAIV use are limited among individuals with severe asthma or active wheezing within the 7 days before vaccination.

## Introduction

Worldwide, the reported prevalence of wheezing illness among children ranges from 7 to 23%; estimates are lower in countries in Asia and Northern/Eastern Europe and higher in North America and Oceania [1]. Because children with a history of wheezing or asthma have a higher risk of complications related to influenza infections [2], the vaccination of children with asthma against seasonal influenza is recommended by the World Health Organization (WHO) and many national health authorities.

In many countries, an Ann Arbor strain live attenuated influenza vaccine (LAIV) is approved for eligible children ≥2 years of age, while some trivalent inactivated influenza vaccines (TIV) are approved for use in eligible children ≥6 months of age. LAIV is not approved for use in children <24 months old because an increase in postvaccination medically attended wheezing was observed between days 7 and 28 after the first vaccination in children aged 6–23 months [3, 4]; this increase was observed among children aged 6–23 months with and without a history of wheezing. In the European Union and Canada, LAIV is approved for use in children ≥2 years of age with mild to moderate asthma or a history of wheezing, with a warning/precaution against use in individuals with severe asthma or active wheezing [5, 6].

The safety and efficacy of LAIV compared with TIV was evaluated in 2,229 children aged 6–17 years with a clinical diagnosis of asthma [7]. In this study, LAIV recipients experienced 35% fewer cases of culture-confirmed influenza illness, with no change in asthma symptomatology compared with children who received TIV. Data regarding the safety and efficacy of LAIV among children aged 6 months to 5 years with asthma or a history of wheezing were collected in the studies by Belshe et al. and Ashkenazi et al., which enrolled healthy children and those with asthma or a history of wheezing [4, 8]. The objective of the current analysis is to present the safety and efficacy data from these two studies for the subgroups of children ≥24 months of age with asthma or a history of wheezing.

## Materials and methods

### Data collection

Safety and efficacy data were collected from two randomized, multinational trials of LAIV and TIV in children aged 6–71 months [4, 8]. In the study by Belshe et al. (study 1), children aged 6–59 months (*N* = 8,475) were randomized 1:1 to receive LAIV or TIV during the 2004–2005 influenza season [4]. Children with mild or moderate asthma or a history of wheezing were included in the study; those with severe asthma or with an episode of wheezing within 42 days before study initiation were excluded. Subjects previously unvaccinated against influenza received two doses of vaccine 28–42 days apart; previously vaccinated subjects received one dose on day 0 (*n*/*N* = 1,899/8,475). Twenty-one percent of subjects (*n*/*N* = 1,793/8,475) reported a history of any wheezing at enrolment; 4% of subjects (*n*/*N* = 338/8,475) reported a diagnosis of asthma. Predefined reactogenicity events (REs) were analyzed for 11 days postvaccination. Adverse events (AEs), including wheezing, were analyzed for 42 days postvaccination. Medically significant wheezing (MSW) was prospectively defined as the presence of wheezing during a physical examination conducted by a healthcare provider with a prescription for a daily bronchodilator, respiratory distress, or hypoxemia; however, other medically attended wheezing episodes were also recorded. Serious AEs (SAEs), including hospitalizations, were analyzed for 180 days after the final dose.

In the study by Ashkenazi et al. (study 2), children aged 6–71 months (*N* = 2,187) were randomized 1:1 to receive two doses of LAIV or TIV 35 ± 7 days apart during the 2002–2003 influenza season [8]. Children enrolled were required to have a history of recurrent respiratory tract infections (RTIs), defined as ≥2 medically attended episodes of common colds, acute otitis media, bronchitis, pneumonia, bronchiolitis, or other RTIs in the previous 12 months; subjects with an episode of a respiratory illness with wheezing within 7 days before study initiation were excluded. Forty-six percent of subjects (*n*/*N* = 1,007/2,187) reported a history of wheezing at enrolment; 23% of subjects (*n*/*N* = 502/2,187) reported a diagnosis of asthma. Predefined REs were recorded for 11 days postvaccination. The incidence of medically documented wheezing was analyzed for 42 days postvaccination; this could not be classified as medically attended wheezing because the protocol mandated office visits for any observed wheezing events. Other AEs were recorded for 28 days postvaccination. SAEs, including hospitalizations, were recorded for the duration of the study, which was approximately 180 days.

### Data analysis

The population for the current analysis was subjects in studies 1 or 2 who were ≥24 months of age with a diagnosis of asthma or a history of wheezing at enrolment. Based on the differences between the studies in data collection, inclusion/exclusion criteria, geography, and influenza season, the two study populations were analyzed separately. In both studies, investigators reported at enrolment whether a child had a prior diagnosis of asthma or had a medical history of wheeze; for those subjects with a reported history of wheeze, investigators stated whether the child had wheezed in the 12 months before enrolment. Additional information was collected regarding past medication use for wheeze or asthma and whether wheeze was documented by a healthcare provider.

The primary endpoints of interest were those related to wheezing events; these endpoints were constructed in order to be consistent with the primary analyses of the original studies while enabling comparison between studies. In both studies, wheezing endpoints were analyzed for 42 days postvaccination in LAIV versus TIV recipients. In study 1, the available wheezing endpoints were any wheezing, medically attended wheezing, and medically significant wheezing; in study 2, the wheezing endpoints available were any wheezing and medically documented wheezing. For the primary endpoints of interest, the analysis population was further stratified into three groups according to medical history at enrolment: (1) children with a history of wheezing/asthma with a wheezing episode within 12 months before study initiation, (2) children with a history of wheezing/asthma but without a wheezing episode in the 12 months before study initiation, and (3) children with a diagnosis of asthma regardless of the timing of their most recent wheezing episode.

Secondary outcomes, which were evaluated in all subjects with a history of wheeze or asthma, included rates of REs for 10 days postvaccination, AEs for 28 days postvaccination, all-cause hospitalization for 180 days, and the relative efficacy of LAIV compared with TIV against culture-confirmed influenza illness during the influenza surveillance periods. AEs were summarized by system organ class and preferred term using Medical Dictionary for Regulatory Activities (MedDRA) version 8.0. Additionally, AEs due to lower respiratory tract illness (LRI) and wheezing were summarized. All reported AEs considered to be related to wheezing, asthma, bronchial obstruction, or bronchospasm (including bronchiolitis) were grouped into wheezing events of special interest for analysis. Lower respiratory AEs included all wheezing events plus any term that referred to disease of the lower respiratory system or to respiratory difficulty.

The safety population, defined as subjects who received ≥1 dose and were followed for safety, was used in analyzing the safety endpoints (wheezing endpoints, REs, AEs, all-cause hospitalizations). Rate differences were calculated as LAIV incidence minus TIV incidence. Two-sided 95% asymptotic confidence intervals (CIs) were computed based on inverting two one-sided tests and the score test statistic. To be consistent with the original studies, relative efficacy calculations used the per-protocol population, generally defined as subjects who did not experience a major protocol violation during the study. The relative efficacy of LAIV compared with TIV and its 95% CI were computed based on the methods used in the original studies.

## Results

### Subject demographics

The analysis population included 1,940 children aged 24–71 months with asthma or a history of wheeze, of whom 1,145 were enrolled in study 1 and 795 were enrolled in study 2. Within each study, the LAIV and TIV treatment groups were well matched for age, gender, race, and wheezing/asthma history (Table 1). Consistent with the study inclusion criteria and site locations, subjects in study 2 were older, more likely to be white, to have wheezed in the last 12 months, and to have been diagnosed with asthma compared with subjects in study 1. For children with a history of wheezing, investigators reported that wheezing had been documented by a healthcare professional in 95% and 98% of patients in studies 1 and 2, respectively. In study 1, LAIV recipients reported less prior inhaled corticosteroid use (Table 2); in study 2, LAIV recipients reported less prior use of systemic corticosteroids. Otherwise, prior medication use was similar between LAIV and TIV recipients in both studies.

**Table 1:** Demographics of children with asthma or a history of wheezing

	Study 1 (Belshe et al. [4])		Study 2 (Ashkenazi et al. [8])	
	LAIV (*N* = 572)	TIV (*N* = 573)	LAIV(*N* = 406)	TIV (*N* = 389)
Age, months, mean (SD)	37 (10)	37 (10)	45 (14)	46 (14)
Gender, *n* (%)				
Male	329 (58)	334 (58)	228 (56)	232 (60)
Female	243 (42)	239 (42)	178 (44)	157 (40)
Race^a^, *n* (%)				
Asian	17 (3)	21 (4)	3 (1)	4 (1)
Black	16 (3)	16 (3)	4 (1)	2 (1)
White	528 (92)	521 (91)	397 (98)	378 (97)
Other	11 (2)	15 (3)	2 (1)	5 (1)
With history of wheeze in last 12 months, *n* (%)	259 (45)	275 (48)	287 (71)	279 (72)
Without history of wheeze in last 12 months, *n* (%)	313 (55)	298 (52)	119 (29)	110 (28)
Diagnosis of asthma, *n* (%)	124 (22)	131 (23)	209 (51)	215 (55)

LAIV: Ann Arbor strain live attenuated influenza vaccine, TIV: trivalent inactivated influenza vaccine

^a^Race was determined by parent/guardian report

**Table 2:** Reported prior medication use for wheeze or asthma by history of wheeze or asthma

	Any history of wheeze/asthma				Diagnosed with asthma			
	Study 1 (Belshe et al. [4])		Study 2 (Ashkenazi et al. [8])		Study 1 (Belshe et al. [4])		Study 2 (Ashkenazi et al. [8])	
	LAIV (*N* = 572)	TIV (*N* = 573)	LAIV (*N* = 406)	TIV (*N* = 389)	LAIV (*N* = 124)	TIV (*N* = 131)	LAIV (*N* = 209)	TIV (*N* = 215)
Medication ever taken for wheeze or asthma, *n* (%)	503 (88)	498 (87)	386 (95)	369 (95)	120 (97)	129 (98)	209 (100)	213 (99)
Short-acting beta-agonist (inhaled or oral), *n* (%)	462 (81)	463 (81)	349 (86)	331 (85)	115 (93)	120 (92)	200 (96)	198 (92)
Long-acting beta-agonist (inhaled or oral), *n* (%)	17 (3)	21 (4)	45 (11)	46 (12)	5 (4)	8 (6)	30 (14)	37 (17)
Cromoglycate and related products, *n* (%)	1 (<1)	10 (2)*	26 (6)	19 (5)	0 (0)	3 (2)	12 (6)	10 (5)
Leukotriene receptor antagonist, *n* (%)	42 (7)	44 (8)	25 (6)	27 (7)	29 (23)	31 (24)	18 (9)	22 (10)
Theophylline, *n* (%)	5 (1)	2 (<1)	12 (3)	10 (3)	2 (2)	1 (1)	8 (4)	6 (3)
Inhaled corticosteroids, *n* (%)	167 (29)	199 (35)**	236 (58)	216 (56)	61 (49)	79 (60)	160 (77)	154 (72)
Systemic corticosteroids, *n* (%)	130 (23)	116 (20)	84 (21)	117 (30)*	39 (32)	42 (32)	51 (24)	80 (37)*
Other asthma medication, *n* (%)	39 (7)	33 (6)	60 (15)	63 (16)	11 (9)	12 (9)	22 (11)	29 (14)

LAIV: Ann Arbor strain live attenuated influenza vaccine, TIV: trivalent inactivated influenza vaccine

*p ≤ 0.01 for LAIV vs. TIV; **p = 0.05 for LAIV vs. TIV

### Rates of wheezing

**Fig. 1:**
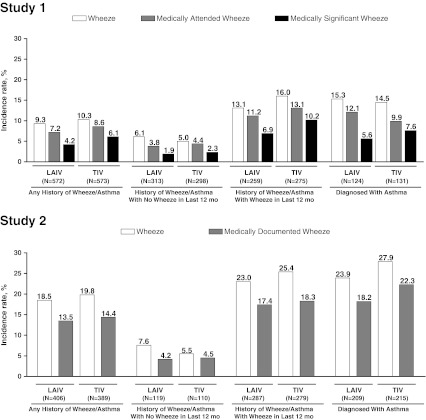
Rates of wheezing for 42 days following vaccination by history of wheeze or asthma. LAIV: live attenuated influenza vaccine, TIV: trivalent inactivated influenza vaccine. There were no statistically significant differences between the incidence of wheezing in LAIV versus TIV recipients

In both treatment groups and in both studies, the rates of wheezing were higher in children with a diagnosis of asthma or wheezing in the last 12 months versus those without wheezing in the last 12 months. Additionally, a higher percentage of subjects in study 2 experienced a wheezing episode after vaccination compared with study 1. In both studies, there were no significant differences in the rates of any wheezing, or medically significant, medically attended, or medically documented wheezing among children receiving LAIV versus TIV overall or when stratified according to medical history (Fig. 1)

### Reactogenicity events

**Fig. 2:**
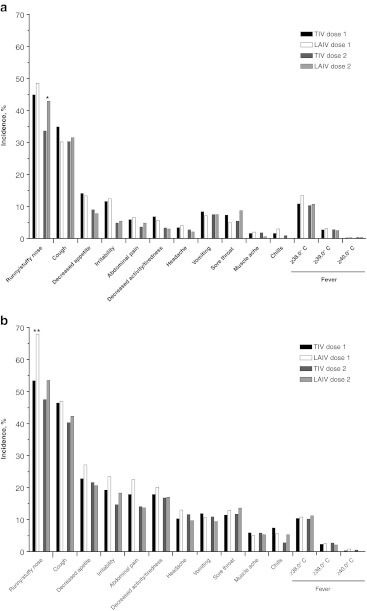
Reactogenicity of LAIV and TIV for 10 days following vaccination by dose. **a** Percentage of subjects experiencing each event in study 1. **b** Percentage of subjects experiencing each event in study 2. LAIV: live attenuated influenza vaccine, TIV: trivalent inactivated influenza vaccine. *p < 0.05, **p < 0.01

The incidence of REs was similar among LAIV and TIV recipients in each study and tended to be less frequent after a second vaccination. The most common REs reported in both studies were runny or stuffy nose and cough (Fig. 2). In both studies, the incidence of runny or stuffy nose was generally higher among LAIV recipients than TIV recipients. The differences were significant after dose 2 in study 1 and after dose 1 in study 2. For other REs, there were no significant differences between LAIV and TIV recipients.

### Adverse events

For both studies, the proportions of subjects reporting ≥1 AE were similar among LAIV and TIV recipients. Similarly, in both studies, there were no differences in the rates of LRI or wheezing for LAIV versus TIV recipients after either dose (Table 3). In study 1, there were no significant differences between LAIV and TIV recipients when events were categorized by organ class or individual terms. In study 2, the rate of events categorized as infections was higher among LAIV recipients after dose 1 (LAIV, 35.2%; TIV, 27.8%; *p* < 0.03). The biggest driver of this difference at the individual term level and the only term with a rate difference ≥1.0% was upper respiratory tract infection (LAIV, 16.5%; TIV, 13.9%; *p* = 0.32). Additionally, in study 2 after dose 1, rhinorrhea (LAIV, 8.1%; TIV, 3.1%; *p* = 0.002) and irritability (LAIV, 2.0%; TIV, 0.3%; *p* = 0.04) were more common among LAIV recipients; presurgical antibiotic prophylaxis was more common among TIV recipients after dose 2 (LAIV, 0%; TIV, 1.6%; *p* = 0.01).

**Table 3:** Rates of adverse events (any, wheezing illness, and lower respiratory illness) for 28 days following vaccination

	Following dose 1			Following dose 2		
	LAIV, *n* (%)	TIV, *n* (%)	Rate difference, %^a^ (95% CI)	LAIV, *n* (%)	TIV, *n* (%)	Rate difference, %^a^ (95% CI)
Study 1						
Subjects, *N*	572	573		335	338	
Total number of events, *n*	294	304		122	145	
Subjects reporting ≥1 events	197 (34.4)	194 (33.9)	0.6 (−4.9 to 6.1)	85 (25.4)	101 (29.9)	−4.5 (−11.3 to 2.3)
Lower respiratory illness	54 (9.4)	61 (10.6)	−1.2 (−4.7 to 2.3)	24 (7.2)	34 (10.1)	−2.9 (−7.3 to 1.4)
Wheezing illness	31 (5.4)	37 (6.5)	−1.0 (−3.9 to 1.7)	9 (2.7)	16 (4.7)	−2.0 (−5.2 to 0.9)
Study 2						
Subjects, *N*	406	389		391	378	
Total number of events, *n*	425	361		339	329	
Subjects reporting ≥1 events	222 (54.7)	195 (50.1)	4.6 (−2.4 to 11.5)	195 (49.9)	185 (48.9)	0.9 (−6.1 to 8.0)
Lower respiratory illness	31 (7.6)	37 (9.5)	−1.9 (−5.9 to 2.0)	41 (10.5)	43 (11.4)	−0.9 (−5.4 to 3.6)
Wheezing illness	21 (5.2)	25 (6.4)	−1.3 (−4.7 to 2.0)	17 (4.3)	19 (5.0)	−0.7 (−3.8 to 2.4)

LAIV: Ann Arbor strain live attenuated influenza vaccine, TIV: trivalent inactivated influenza vaccine

^a^Rate difference = LAIV rate − TIV rate; none of the rate differences are statistically significant

### Hospitalizations

There were few hospitalizations in the populations studied. In study 1, there were 25 hospitalizations in 25 subjects for 180 days after vaccination; in study 2, there were 36 hospitalizations in 32 subjects. In both studies, there were no significant differences in the incidence of hospitalization among children who received LAIV and TIV. In study 1, hospitalization rates were 2.4% among LAIV recipients and 1.9% among TIV recipients, for a rate difference of 0.5% (95% CI −1.2 to 2.4). In study 2, hospitalization rates were 4.7% among LAIV recipients and 3.3% among TIV recipients, for a rate difference of 1.3% (95% CI −1.5 to 4.2). Hospitalization rates within 90 days after vaccination were also similar for each treatment group in each study (study 1: LAIV, 1.2%; TIV, 1.4%; study 2: LAIV, 3.4%; TIV, 3.3%). A review of all hospitalization diagnoses demonstrated that, in study 1, there were ten hospitalizations due to LRI (e.g., pneumonia, croup, wheezing, bronchospasm; LAIV, *n* = 6; TIV, *n* = 4), five of which occurred within 90 days after vaccination (LAIV, *n* = 2; TIV, *n* = 3). In study 2, there were 18 hospitalizations due to LRI (LAIV, *n* = 11; TIV, *n* = 7), 15 of which occurred within 90 days after vaccination (LAIV, *n* = 8; TIV, *n* = 7).

### Relative efficacy

**Fig. 3:**
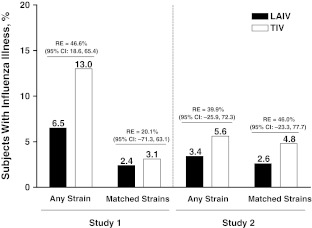
Relative efficacy of LAIV and TIV in children aged 2–5 years with a history of wheeze or asthma. Any strain refers to any community-acquired strain. LAIV: live attenuated influenza vaccine, TIV: trivalent inactivated influenza vaccine, RE: relative efficacy, CI: confidence interval

In studies 1 and 2, the relative efficacy of LAIV versus TIV among children with a history of wheezing or asthma was consistent with that observed in the overall study cohorts. In all instances, children receiving LAIV had fewer cases of culture-confirmed influenza illness than TIV recipients (Fig. 3). In study 1, for all community-acquired strains, the relative efficacy of LAIV versus TIV reached statistical significance (relative efficacy, 46.6%; 95% CI 18.6 to 65.4), despite the small number of subjects in the analysis population. In study 2, the difference was not statistically significant.

## Discussion

The Ann Arbor strain LAIV has been evaluated in multiple randomized controlled studies in children with a medical history of wheezing and asthma. The first study evaluated the effect on pulmonary function, as measured by spirometry, in children aged 9 years and older with moderate to severe asthma. In this small study (*N* = 48), no changes in pulmonary function were detected after vaccination with LAIV versus placebo [9].

Subsequently, three large, randomized, TIV-controlled studies were conducted in children with a history of asthma or wheezing to further characterize the clinical outcomes after receipt of LAIV. Collectively, these studies included 4,169 children aged 2–17 years who were diagnosed with asthma or had a medically confirmed history of wheezing. All of the studies were prospectively designed to evaluate the effect of LAIV on asthma exacerbations and/or wheezing. The first study by Fleming et al. compared LAIV and TIV in 2,229 children aged 6–17 years with a clinical diagnosis of asthma [7]. The study demonstrated that children who received LAIV experienced 35% fewer cases of culture-confirmed influenza illness, with asthma exacerbation rates, mean peak expiratory flow rate findings, asthma symptom scores, or nighttime awakening scores that were similar to TIV recipients.

The current analysis provides the data from two studies regarding the safety and efficacy of LAIV in children aged 2–5 years with a history of asthma or wheezing. Similar to the results observed by Fleming et al., there were no statistically significant increases in wheezing, LRI, or hospitalization among children receiving LAIV versus TIV. The type and incidence of REs and AEs observed in this study were consistent with those observed after vaccination with LAIV and TIV in other studies of young children. As expected, a higher rate of runny nose/nasal congestion was observed among LAIV recipients; this difference also likely explains the few unsolicited AEs that were increased among LAIV recipients (rhinorrhea and upper respiratory tract infection). These findings are supported by the results of a large, nonrandomized study of LAIV conducted over 4 years that included 2,196 healthy children aged 18 months to 18 years with a history of intermittent wheezing or asthma and found no increased risk for medically attended acute respiratory illness or asthma after vaccination with LAIV [10].

In the original analysis of Belshe et al.’s study [4], a post hoc trend toward an increased rate of all-cause hospitalization was observed among children aged 6–47 months, but not among those aged 48–59 months with a history of any prior wheezing. Following additional review of the data, officials at the U.S. Food and Drug Administration concluded that, for children ≥24 months of age who received LAIV, “there was little influence of history of wheezing on hospitalization and the rates of hospitalization were either similar to or actually higher in the TIV arm” and stated that the relationship of history of wheezing to a potential risk of all-cause hospitalization was likely “a statistical blip” [11]. Additionally, no such trend was observed in Ashkenazi et al.’s study [8]. The current analysis, which incorporates data from two distinct studies, provides a more comprehensive view of the safety of LAIV in young children with asthma or a history of wheezing.

Because LAIV vaccination entails nasopharyngeal replication of attenuated influenza viruses, some may raise concern regarding the long-term effects on airway function. However, it is rhinovirus-induced wheezing in the first 2–3 years of life, and not illness due to influenza, that has been correlated with allergic sensitization and subsequent asthma pathogenesis [12, 13]. Furthermore, a recent study demonstrated that allergic sensitization to aeroallergens precedes rhinovirus-induced wheezing, and viral wheeze did not lead to subsequent allergic sensitization [14]. The current analysis, the study by Fleming et al., and multiple additional studies conducted in young children without asthma demonstrate that LAIV vaccination in children 2 years of age and older is not associated with subsequent wheezing or lower respiratory illness [7, 15]. In fact, in children aged 6–17 years with asthma, there was an 18% reduction (*p* = 0.02) in wheezing in the 15 days after vaccination in LAIV versus TIV recipients [7].

It should be noted that the safety of Ann Arbor strain LAIV has not been extensively studied among severe asthmatics (e.g., individuals currently requiring therapy with oral glucocorticosteroids or high-dose inhaled glucocorticosteroids) or individuals with recent active wheezing within the 7 days before vaccination. Additionally, the high rates of postvaccination wheezing in both treatment groups also highlight the difficulty of using non-controlled, passively collected surveillance data to evaluate vaccine safety in children with asthma or a history of wheezing [16].

Both studies analyzed demonstrated fewer cases of culture-confirmed influenza illness among LAIV versus TIV recipients in the original overall study populations [4, 8]. The current analysis demonstrates consistent results among the cohorts of children with a history of asthma/wheezing, with one analysis reaching statistical significance despite the small study population. These data support the conclusion that Ann Arbor strain LAIV continues to provide a high level of protection against influenza illness in young children with asthma or a history of wheezing. The efficacy of the vaccine in this subset of children is important because annual vaccination against seasonal influenza is generally recommended in this population.

Strengths of the current analysis include the randomization of study subjects to LAIV versus TIV that provided two treatment groups with similar baseline characteristics, the prospective collection of history of asthma and wheezing at enrolment, and the prospective definitions of the wheezing endpoints. The analysis also benefits from the ability to analyze children of similar age from two separate studies conducted in different influenza seasons. The primary limitation of the current analysis is the post hoc definition of the study cohorts, but this was addressed in part by the analysis of the three subpopulations that varied based on the severity and recency of wheezing illness. Additionally, the identification of subjects with a diagnosis of asthma or history of wheezing relied on investigator judgment; there was no validation of investigator assessments. However, the proportions of subjects with prior receipt of inhaled and systemic corticosteroids help to validate that the identified children had significant wheezing disease.

In conclusion, in the European Union and Canada, LAIV is currently approved for use among children aged 2–17 years, including those with mild to moderate asthma or a history of wheezing. The results of the current analysis and previous studies support the safety and efficacy of Ann Arbor strain LAIV in these populations.
